# Connexin32 regulates hepatoma cell metastasis and proliferation via the p53 and Akt pathways

**DOI:** 10.18632/oncotarget.2687

**Published:** 2014-11-27

**Authors:** Bixing Zhao, Wenxiu Zhao, Yu Wang, Yaping Xu, Jianfeng Xu, Kai Tang, Sheng Zhang, Zhenyu Yin, Qiao Wu, Xiaomin Wang

**Affiliations:** ^1^ Department of Hepatobiliary Surgery, Zhongshan Hospital, Xiamen University. Fujian Provincial Key Laboratory of Chronic Liver Disease and Hepatocellular Carcinoma, Xiamen University Affiliated Zhongshan Hospital; ^2^ Research Institute of Digestive Disease, Xiamen University, Xiamen, Fujian, China; ^3^ State Key Laboratory of Cellular Stress Biology, Innovation Center for Cell Biology, School of Life Sciences, Xiamen University, Xiamen, Fujian Province, China

**Keywords:** connexin32, p53, hepatocellular carcinoma, invasion, migration

## Abstract

Hepatocellular carcinoma (HCC) progresses rapidly and is frequently associated with vascular invasion, metastasis, recurrence, and poor prognosis. The expression of connexin32 (Cx32) is frequently downregulated in HCC tissues. In this study, the role of Cx32 in HCC metastasis and proliferation was investigated. The reduction of Cx32 in HCC tissues was significantly associated with increased vascular invasion, increased tumor size, and poor survival. *In vitro* assays revealed that Cx32 not only suppressed the invasion and migration of HCC cells, but also repressed HCC cell proliferation. Subsequent investigations revealed that Cx32 directly enhanced the acetylation and transcriptional activity of p53, thus upregulating the expression of the tumor metastasis suppressor protein KAI1/CD82, which is a p53 target gene. Additionally, Cx32 negatively regulated the phosphorylation of Akt and the expression of the cell cycle regulation protein cyclin D1, thereby inhibiting the proliferation of HCC cells. Our *in vivo* nude mice model further confirmed that Cx32 is able to suppress HCC tumor growth and metastasis in nude mice. Our results imply that Cx32 downregulation contributes to the proliferation and metastasis of HCC, and the restoration of Cx32 expression may be a promising strategy for HCC therapy.

## INTRODUCTION

HCC is the fifth most common human cancer and the third leading cause of cancer death worldwide [1]. Frequent metastasis is responsible for the rapid recurrence of HCC and the poor survival in affected patients. However, the molecular mechanisms underlying HCC development and metastasis have not been elucidated.

Recently, molecularly targeted drugs for HCC treatment have been extensively studied. Multiple molecular pathways are implicated in HCC pathogenesis, including pathways involving vascular endothelial growth factor receptor (VEGFR), fibroblast growth factor receptor (FGFR), platelet-derived growth factor receptor (PDGFR), epidermal growth factor receptor (EGFR) [2], and hepatocyte growth factor (HGF)/c-MET [3] and the PI3K/AKT/mTOR pathway [4]. An increasing number of HCC-related signaling pathways may act as potential targets for therapeutic interventions. For example, Sorafenib, which is the only standard drug used for the treatment of patients with advanced HCC, is an oral multi-kinase inhibitor that blocks multiple growth factor pathways, including those involving VEGFR-1, -2, -3, PDGFR-β, Raf, RET, and FLT-3 [5].

Gap junction channels, which are located at cell-cell contact sites, are composed of connexins (Cxs) and mediate the inter-cellular flux of metabolites, nutrients, and secondary messengers [6, 7]. Gap junction intercellular communication and Cxs play important roles in organ/tissue homeostasis and cell differentiation [6, 8]. Individual Cxs are defined and named based on their molecular weight and differ both in terms of function and expression patterns [9]. Cx32 and Cx26 are the main gap junction proteins in hepatocytes [6]. A previous study showed evidence that strongly supports the hypothesis that Cx genes act as tumor suppressor genes [10]. However, newer data now suggest that they may also play a role in tumor progression. Several reports suggest that Cxs might facilitate invasion, intravasation, extravasation, and metastasis [11–13]. Thus, studies examining the relationship between Cxs and tumor cell invasion have been controversial, and the findings should be clarified further.

Abnormal cell proliferation, which results from the deregulation of the cell cycle, forms the crux of the cancer phenotype. Accumulating evidence has clearly demonstrated a role for Cxs in cell proliferation. A decrease in cell proliferation was commonly observed in earlier studies that focused on the phenotypic changes in cancer cells following enhanced Cx expression [14]. One recent study verified that Cxs play an anti-proliferative role in various cancers, including HCC [15], gastric cancer [16], insulinomas [17], canine bone tumors [18], and non-small cell lung cancer [19]. Therefore, Cxs provide a unique perspective when studying specific aspects of cell cycle regulation, and these insights may help develop more focused therapies that target this process.

In this study, we demonstrate the significance of Cx32 in tumor cell proliferation and metastasis. First, we found that the downregulation of Cx32 in human HCC tissues was associated with increased tumor sizes, metastasis, and poor survival. *In vitro* and *in vivo* assays showed that Cx32 significantly suppressed HCC proliferation and metastasis. Additionally, we provided further evidence to support the notion that Cx32 exerts its anti-proliferative and anti-metastatic effects via the PI3K/Akt and p53 pathways, respectively.

## RESULTS

### Downregulation of Cx32 is associated with a poor prognosis

**Figure 1:**
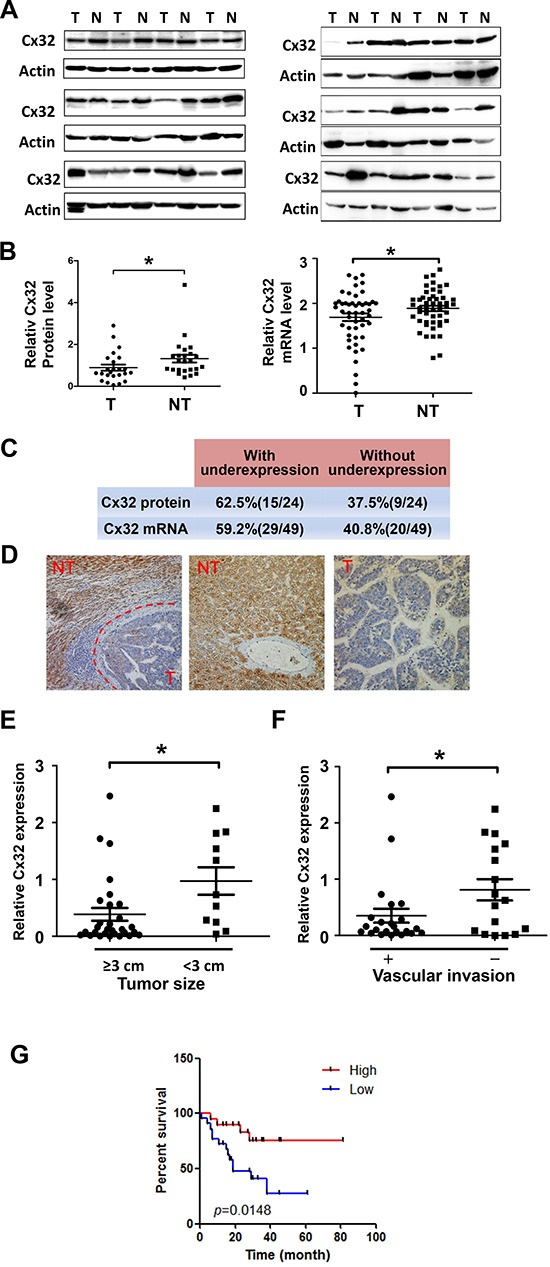
Downregulation of Cx32 in HCC tissues is associated with a poor prognosis for HCC patients **(A)** Western blot analysis of Cx32 protein expression in 24 human HCC samples and their adjacent non-tumorous liver tissue samples. N: non-tumorous liver tissue, T: tumor tissue. **(B)** Quantitative analysis of Cx32 protein expression in 24 human HCC samples and mRNA level analysis of Cx32 in another set of 49 HCC tumor samples (T) vs. non-tumor samples (NT); *p < 0.05. **(C)** Summary of the differences in the expression of Cx32 protein and mRNA between paired tumor and non-tumor liver tissues. **(D)** Immunohistochemical staining for Cx32 in HCC tumor tissue (T) and non-tumorous liver tissue (NT). **(E)** Tumor size was inversely correlated with Cx32 mRNA expression in HCC tissues. The median expression value of all 40 cases was chosen as the cutoff value for separating the dataset into a Cx32–low expression group and a Cx32–high expression group. **(F)** Metastatic HCC displayed lower Cx32 expression levels. The absence (*n* = 17) and presence (*n* = 23) of vascular invasion (tumor thrombus in the veins of adjacent non-tumor tissues or in the portal vein) is indicated with a minus sign (–) and plus sign (+), respectively; *p < 0.05. **(G)** Kaplan-Meier curves revealed an association of lower Cx32 levels with a shorter overall postoperative survival.

Western blotting was first performed to examine the expression of Cx32 in 24 pairs of HCC specimens and adjacent non-tumorous liver samples (Fig. 1A). Quantitative analyses of Cx32 protein expression showed that compared to paired non-tumor tissues, 62.5% of HCC samples showed downregulated levels of Cx32 expression (Fig. 1C); there was a significant difference in relative Cx32 protein levels between paired tumor and non-tumor tissues (*p* = 0.034, Paired *t*-test; Fig. 1B). The Cx32 mRNA expression levels were further examined by quantitative real-time PCR in another set of 49 human HCC specimens. Similarly, Cx32 mRNA levels were also downregulated in HCC specimens (*p* = 0.0373, Paired *t*-test; Fig. 1B, 1C). The results of the immunohistochemical analysis of Cx32 expression in sections from paraffin-embedded HCC samples were consistent with those derived from the real-time PCR and western blot analyses (Fig. 1D). Therefore, the downregulation of Cx32 observed in HCC specimens suggests that Cx32 may be involved in HCC progression.

To understand the significance of Cx32 in HCC better, we analyzed the correlation between Cx32 mRNA levels and the clinical features of the HCC patients evaluated in this study (Table 1); the total number of cases used in the statistical analyses was 40, owing to incomplete information on some patients. The median expression value of all 40 cases was chosen as the cutoff value for separating the dataset into a Cx32–low expression group and a Cx32–high expression group [20]. Kaplan-Meier analysis revealed an association between lower Cx32 expression levels and a shorter overall survival time (Fig. 1G). Importantly, lower Cx32 expression levels were significantly associated with large tumor size and vascular invasion (Table 1 & Fig. 1E, 1F). Together, our findings suggest that Cx32 downregulation may contribute to HCC progression by promoting tumor growth and metastasis.

**Table 1:** Correlation of Cx32 mRNA expression with clinicopathological features in hepatocellular carcinoma

Variables	Category	Number of Case	*P* Vlaue
**Age**	<59	28	0.3759
	≥59	12	
**Gender**	Male	34	0.7956
	Female	6	
**Tumor size**	≥3 cm	29	0.0169*
	<3 cm	11	
**Vascular invasion**	Yes	23	0.0395*
	No	17	
**AFP (ng/ml)**	>200	16	0.3667
	≤200	24	
**HBsAg**	Negative	6	0.4716
	Positive	34	
**Cirrhosis**	Absent	11	0.3595
	Present	29	
**TNM stage**	I-II	5	0.2056
	III-IV	35	

Abbreviation: AFP, alpha-fetoprotein.

* *P* < 0.05.

### Cx32 suppresses HCC cell migration and invasion

**Figure 2:**
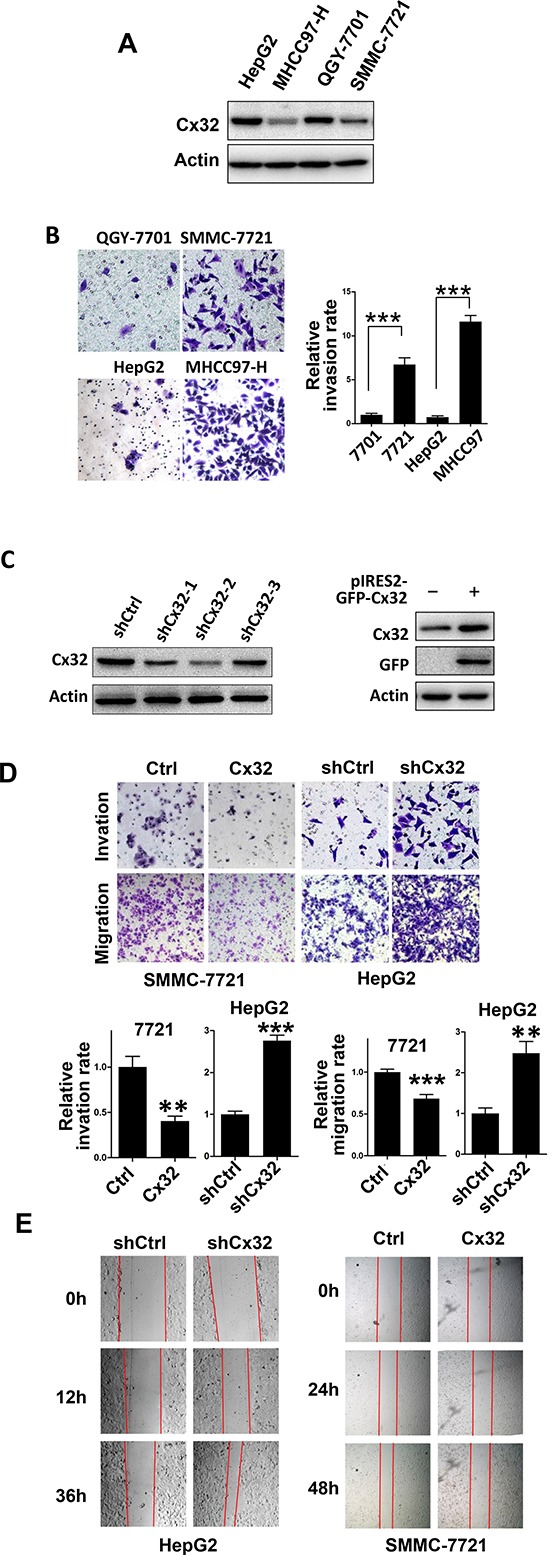
Cx32 represses HCC cell invasion and migration **(A)** Western blot analysis of Cx32 protein expression in one hepatocyte cell line (L-O2) and four human HCC cell lines (HepG2, MHCC97-H, QGY-7701, and SMMC-7721). **(B)** Matrigel invasion assays of HepG2, MHCC97-H, QGY-7701, and SMMC-7721 cells. **(C)** Western blot showing a marked reduction of Cx32 expression in knockdown HepG2 cells, and upregulation of Cx32 in SMMC-7721 cells transfected with the pIRES2-GFP-Cx32 expression vector. **(D)** Overexpression of Cx32 reduces SMMC-7721 cell invasion and migration; downregulation of Cx32 promotes HepG2 cell invasion and migration. **(E)** Wound-healing assay showing that Cx32 inhibited the migration of SMMC-7721 cells and that downregulation of Cx32 promoted the migration of HepG2 cells.

To examine the expression of Cx32 in HCC cells further, a western blot analysis was performed in several HCC cell lines (HepG2, QGY-7701, SMMC-7721, and MHCC97-H) (Fig. 2A). Cx32 protein levels were significantly higher in the HepG2 and QGY-7701 cells than in the MHCC97-H and SMMC-7721 cells, and the metastatic potential of the MHCC97H and SMMC-7721 cells was remarkably greater than that of the HepG2 and QGY-7701 cells (Fig. 2B). Therefore, we hypothesized that Cx32 may negatively regulate the migratory and invasive abilities of HCC cells.

To establish stable Cx32 knockdown cells, HepG2 cells were stably transfected with the pU6 (shCtrl) control vector or the pU6-Cx32-shRNA (shCx32) plasmid. Simultaneously, SMMC-7721 cells were transiently transfected with Cx32/pIRES2-EGFP to increase Cx32 expression. Cx32 expression in each cell line was demonstrated by a western blot analysis (Fig. 2C). shCx32-2, which was shown to result in a significant Cx32 knockdown, was used in subsequent experiments. To elucidate the role of Cx32 in HCC metastasis, the effects of Cx32 on the migration and invasiveness of HCC cells were analyzed. Transwell assays showed that both the migratory and invasive activities of HCC cells were suppressed by Cx32 overexpression, but were promoted by cellular Cx32 depletion (Fig. 2D). The migration rate was also measured using a wound healing assay, and the results confirmed that Cx32 negatively regulates the migration rate of HCC cells (Fig. 2E).

Together, these results indicate the suppressive effects of Cx32 on HCC migration and invasiveness.

### Cx32 represses HCC metastasis via the p53 pathway

**Figure 3:**
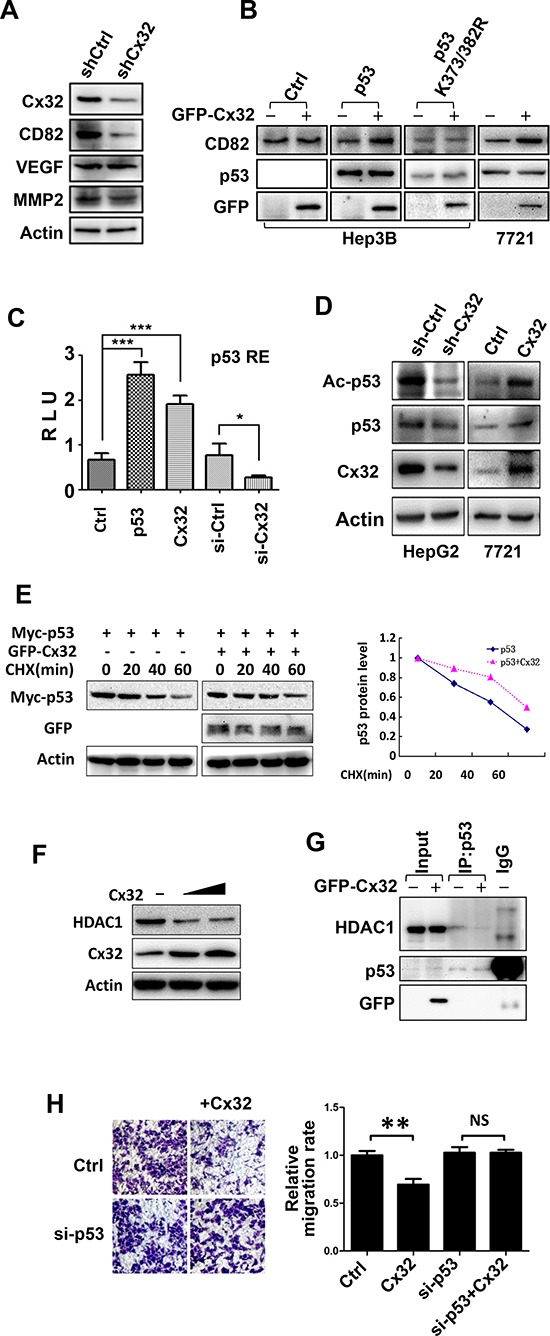
Cx32 exerts its anti-metastatic function via the p53-CD82 pathway **(A)** The protein level of CD82 was downregulated in shCx32 HepG2 cells. **(B)** Cx32 upregulated the expression of CD82 in a p53-dependent manner. Cx32, p53, or p53 mutant (K373/382R) expression vectors were transfected into Hep3B or SMMC-7721 cells, as indicated, and the cells were monitored for the expression of CD82 by western blot. **(C)** Cx32 induced p53 transcriptional activity. The p53-Luciferase reporter and β-galactosidase (β-gal) gene expression vectors, together with p53, Cx32 expression vectors, Cx32 siRNA, or siCtrl, as indicated, were transfected into 293T cells. Reporter gene activity was determined and normalized in relation to the co-transfected β-gal activity. The bars represent the mean ± SEM from three independent experiments. **(D)** Cx32 positively regulated p53 acetylation. Cx32 knockdown HepG2 cells and control cells were collected, and SMMC-7721 cells were non-transfected or transfected with the Cx32 expression vector for 48 h. Each cell lysate was then subjected to western blot analysis using an anti-Acetylated-p53 (K373/382), -Cx32, or -p53 antibody. **(E)** Cx32 prolongs the half-life of p53. Cx32 and Myc-p53 were transfected into 293T cells and then treated with CHX (100 μg/ml) for the indicated times. p53 expression level was determined by western blotting using an anti-Myc antibody. The levels of p53 protein were quantified by densitometry. **(F)** Cx32 inhibited HDAC1 expression in a dose-dependent manner. **(G)** Cx32 inhibited the interaction of p53 and HDAC1. GFP-Cx32 was transfected into SMMC-7721 cells, as indicated. The loading of HDAC1 was normalized before immunoprecipitation, and the cell lysate was then immunoprecipitated using an anti-p53 antibody. The immunoprecipitates were examined by western blotting using an anti-HDAC1 antibody. The input represented 10% of the cell lysates used in the co-IP experiment. **(H)** p53 siRNA attenuated the anti-migratory function of Cx32. SMMC-7721 cells were transfected with Cx32, siCtrl, or si-p53, as indicated. Twenty-four hours later, cells were added to transwell chambers and incubated for 20 h, followed by staining with crystal violet; *p < 0.05; **p < 0.01; ***p < 0.001.

To understand the mechanisms underlying the changes in the invasive and migratory abilities of HCC cells, the levels of several invasion-related proteins were compared between the control HepG2 cells and the Cx32-depleted HepG2 cells, using western blot analysis. Downregulation of Cx32 did not cause a significant change in the expression of matrix metallopeptidase 2 (MMP2) or in that of VEGF, but did result in a significant decrease in the expression of KAI1/CD82, a tumor metastasis suppressor protein (Fig. 3A). The expression of CD82 in SMMC-7721 cells was increased following Cx32 overexpression (Fig. 3B). However, in the p53-null Hep3B cells, overexpression of Cx32 failed to alter the expression of CD82 (Fig. 3B). Therefore, we believe that the upregulation of CD82 expression by Cx32 is p53-dependent because KAI1/CD82 transcription is also upregulated by p53 [21]. To confirm this notion, p53 was co-expressed in Hep3B cells; under these conditions, Cx32 was able to upregulate CD82 expression (Fig. 3B).

Because p53 is the transcription activator of KAI1/CD82, Cx32 may lead to CD82 upregulation by merely increasing the transcriptional activity of p53. We investigated this possibility by using a p53-luciferase reporter system. As shown in Fig. 3C, Cx32 significantly induced the activity of the p53 reporter gene, and the addition of Cx32 siRNA significantly reduced p53 transcriptional activity.

It has been reported that acetylation of p53 by p300/CBP on multiple lysine residues leads to the activation of p53 transcriptional activity [22]. Thus, it is possible that Cx32 affects p53 acetylation, leading to the induction of p53 transcriptional activity. To test this possibility, the acetylation level of p53 was tested using an antibody specific for p53 acetylation at K373/K382. As expected, Cx32 knockdown greatly decreased the acetylation level of p53, while Cx32 overexpression significantly induced p53 acetylation (Fig. 3D). We concluded that Cx32 could enhance p53 acetylation, which might contribute to the upregulation of p53 transcriptional activity, resulting in the upregulation of the downstream protein CD82.

Next, we confirmed our hypothesis by using a unique p53 point mutant, p53K373/K382R. When this point mutant was transiently transfected into Hep3B cells, the introduction of Cx32 could not alter the expression level of CD82 in relation to the wild-type p53 group (Fig. 3B). Thus, this result showed that the Cx32-mediated induction of the CD82 protein is dependent on p53 acetylation.

One consequence of p53 acetylation is a decrease in p53 degradation [23]. To evaluate the effect of Cx32 on p53 protein stability, SMMC-7721 cells were treated with cycloheximide (CHX), an inhibitor of protein synthesis. Western blot analysis (Fig. 3E) indicated that Cx32 significantly prolongs the half-life of p53.

To understand the mechanism by which Cx32 affects p53 acetylation, we examined histone deacetylase 1 (HDAC1), which directly regulates the deacetylation of p53. Previous studies have shown that restoration of Cx32 expression in Cx32-deficient hepatocytes results in a 2.5-fold decrease in the expression of HDAC1 [24]. We also observed that Cx32 downregulated the level of HDAC1 protein in a dose-dependent manner in SMMC-7721 cells (Fig. 3F).

It has been reported that interactions between p53 and HDACs result in p53 deacetylation, thereby reducing p53 transcriptional activity [25]. We wondered whether Cx32 induces p53 acetylation and transcriptional activity by affecting the p53-HDAC1 interaction. To confirm this hypothesis, SMMC-7721 cells were transiently transfected with GFP-Cx32, and co-immunoprecipitation (co-IP) assays were performed. Because the introduction of Cx32 results in a decrease in HDAC1 protein levels, HDAC1 protein levels were normalized before the co-IP assay was performed. As expected, HDAC1 was detected in the p53 immunoprecipitate but not in the IgG immunoprecipitate, and transfection of Cx32 significantly suppressed the interaction between p53 and HDAC1 (Fig. 3G).

We next examined whether the negative regulatory effect of Cx32 on HCC cell metastasis was also dependent on p53. SMMC-7721 cells, which harbor wild-type p53 [26], were transiently transfected with p53 siRNA. As shown in Fig. 3H, Cx32 alone was able to inhibit cell migration. However, the addition of p53 siRNA drastically attenuated the inhibition of cell migration caused by Cx32.

These results confirmed the role of Cx32-p53-CD82 cross talk in the regulation of HCC cell migration and invasiveness.

### Cx32 suppresses HCC cell proliferation

**Figure 4:**
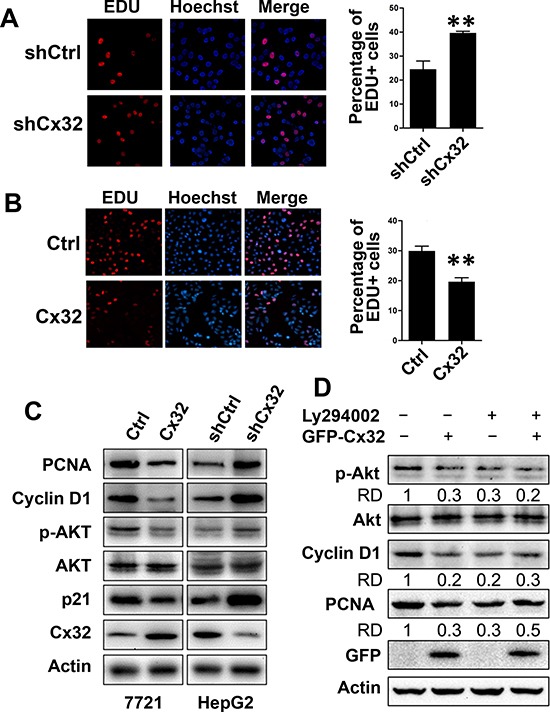
Cx32 suppresses HCC cell proliferation through inhibition of the Akt signaling pathway **(A, B)** EdU assay analysis of the Cx32 knockdown (A) and overexpression (B) effect on the proliferation of HepG2 and SMMC-7721 cells. Cells were cultured in 24-well microtiter plates after transfection or non-transfection. EdU (100 μM) was added, and the cells were cultured for another 2 h before EdU and Hoechst staining. The total cell number and EdU-positive cell number were counted in five random fields; ** *p* < 0.01. **(C)** The expression of PCNA, Akt, and cell cycle regulatory proteins in Cx32-overexpression or knockdown HCC cells and control cells. **(D)** The PI3K inhibitor attenuated the inhibitory function of Cx32 on PCNA and cyclin D1 protein levels, as examined by western blotting. Data were measured as relative density (RD) of p-Akt/Akt, Cyclin D1/Actin, and PCNA/Actin. The first control lane was defined as 1.

To investigate the role of Cx32 in HCC cell proliferation, an EdU assay was performed in stable Cx32-knockdown HepG2 cells and control HepG2 cells. As shown in Fig. 4A, Cx32-knockdown HepG2 cells (sh-Cx32) had a significantly higher positive rate for EdU incorporation than shCtrl HepG2 cells did (39.6% vs. 24.5%, respectively, *p* = 0.0025). Similarly, Cx32 overexpression in SMMC-7721 cells significantly suppressed cell proliferation (from 30% to 19.6% EdU-positive cells, respectively, *p* = 0.0078; Fig. 4B). The expression of the proliferation marker proliferating cell nuclear antigen (PCNA) was also decreased following Cx32 overexpression, and was induced in Cx32-knockdown cells, as determined by western blot analysis (Fig. 4C). These results demonstrate the suppressing effect of Cx32 on HCC cell proliferation. Surprisingly, the expression of the cell cycle inhibitor p21^Cip1/Waf1^ was also decreased in the Cx32-overexpressing SMMC-7721 cells. p21 is a p53 target gene, and Cx32 was shown to positively regulate the transcriptional activity of p53 (Fig. 3C); however, here it negatively regulated p21 expression. Therefore, we concluded that the effect of Cx32 on p21 expression was p53-independent and did not occur at the transcriptional level; thus, p21 might not be involved in the regulation of HCC proliferation by Cx32.

It is well known that Akt/PKB functions as a critical regulator of cell survival and proliferation, and that cyclin D1 is one of the most important regulatory proteins in cell cycle progression and can be modulated by the PI3K/Akt pathway [27]. Therefore, we examined the effects of Cx32 on the activation of Akt signaling and on cyclin D1 expression, by measuring the levels of phosphorylated Akt and cyclin D1. Western blot analysis showed that the expression of cyclin D1 and phosphorylated Akt was significantly decreased when Cx32 was overexpressed in cells and was increased in Cx32-depleted cells (Fig. 4C).

These data indicate that Cx32 suppresses HCC proliferation through its ability to inhibit the phosphorylation and activity of Akt, and the expression of the cell cycle regulatory protein cyclin D1. This hypothesis was further supported by our results that showed that treatment with the PI3K/Akt inhibitor LY294002 drastically attenuated Cx32-mediated inhibition of cyclin D1 and PCNA expression (Fig. 4D). As shown in Figure 4D, transfection of Cx32 impaired Akt phosphorylation and the expression of cyclin D1 and PCNA, while in the LY294002 treatment group, Cx32 did not impair cyclin D1 and PCNA levels. Taken together, the results of the series of experiments described above demonstrated that Cx32 negatively regulated HCC cell proliferation via the Akt signaling pathway.

### Cx32 suppresses HCC progression *in vivo*

**Figure 5:**
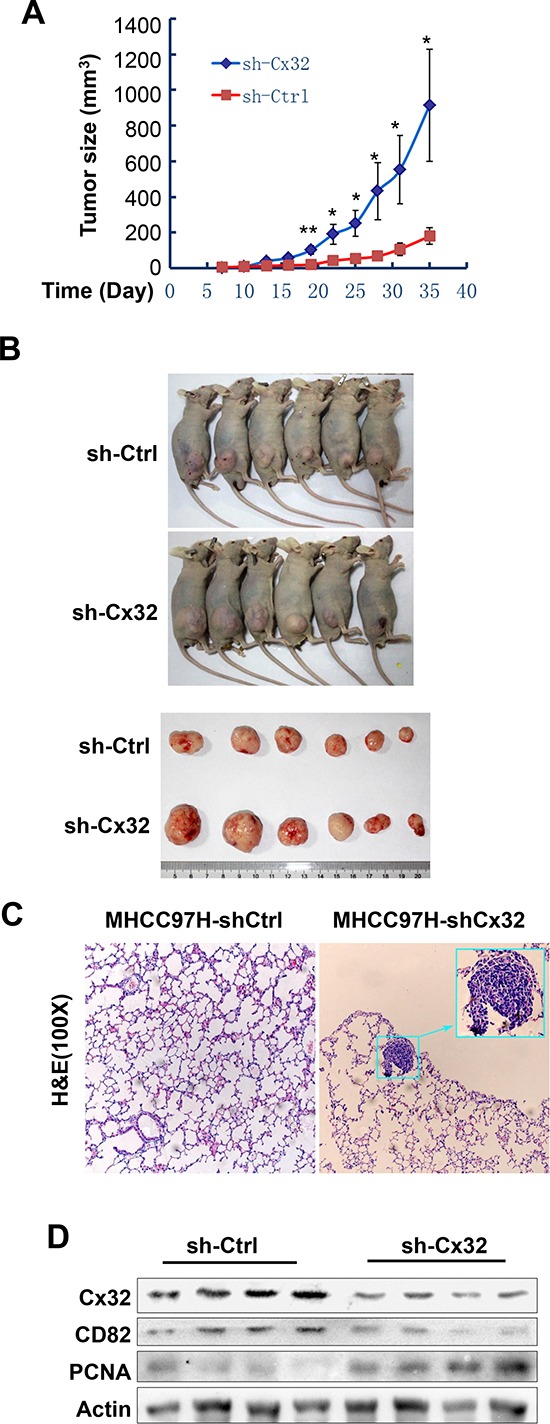
Cx32 suppresses HCC growth and pulmonary metastasis in mouse models **(A)** Growth curves of the MHCC97H-shCtrl and MHCC97H-shCx32 models. **(B)** Macrograph of mice and tumors in both groups. **(C)** Hematoxylin and eosin (H&E)-stained images of metastatic nodules in lungs. **(D)** Western blot analysis of the expression of Cx32, CD82, and PCNA protein in MHCC97H-shCx32 tumors and control tumors; *p < 0.05 and **p < 0.01.

To further investigate whether Cx32 suppresses HCC progression *in vivo*, Cx32 was stably knocked down in the highly metastatic HCC cell line MHCC97H. After subcutaneous transplantation of the MHCC97H-shCtlr and MHCC97H-shCx32 cell lines into nude mice, both groups successfully formed tumors 1 week later. The tumors of the MHCC97H-shCx32 model grew faster than those of the MHCC97H-shCtrl model (Fig. 5A, 5B). Significant differences were observed 19 days after transplantation (*p* < 0.01). Pulmonary metastasis was observed in MHCC97H-shCx32 mice, but not in the control group (Fig 5C). To correlate the biological response with the mechanisms identified in the cells, CD82 and PCNA protein levels were assessed by western blot analysis. As shown in Figure 5D, knockdown of Cx32 significantly suppressed the expression of CD82 and enhanced the expression of PCNA in transplanted tumor tissue. These data indicate that Cx32 was able to suppress HCC tumor growth and metastasis in nude mice.

## DISCUSSION

**Figure 6:**
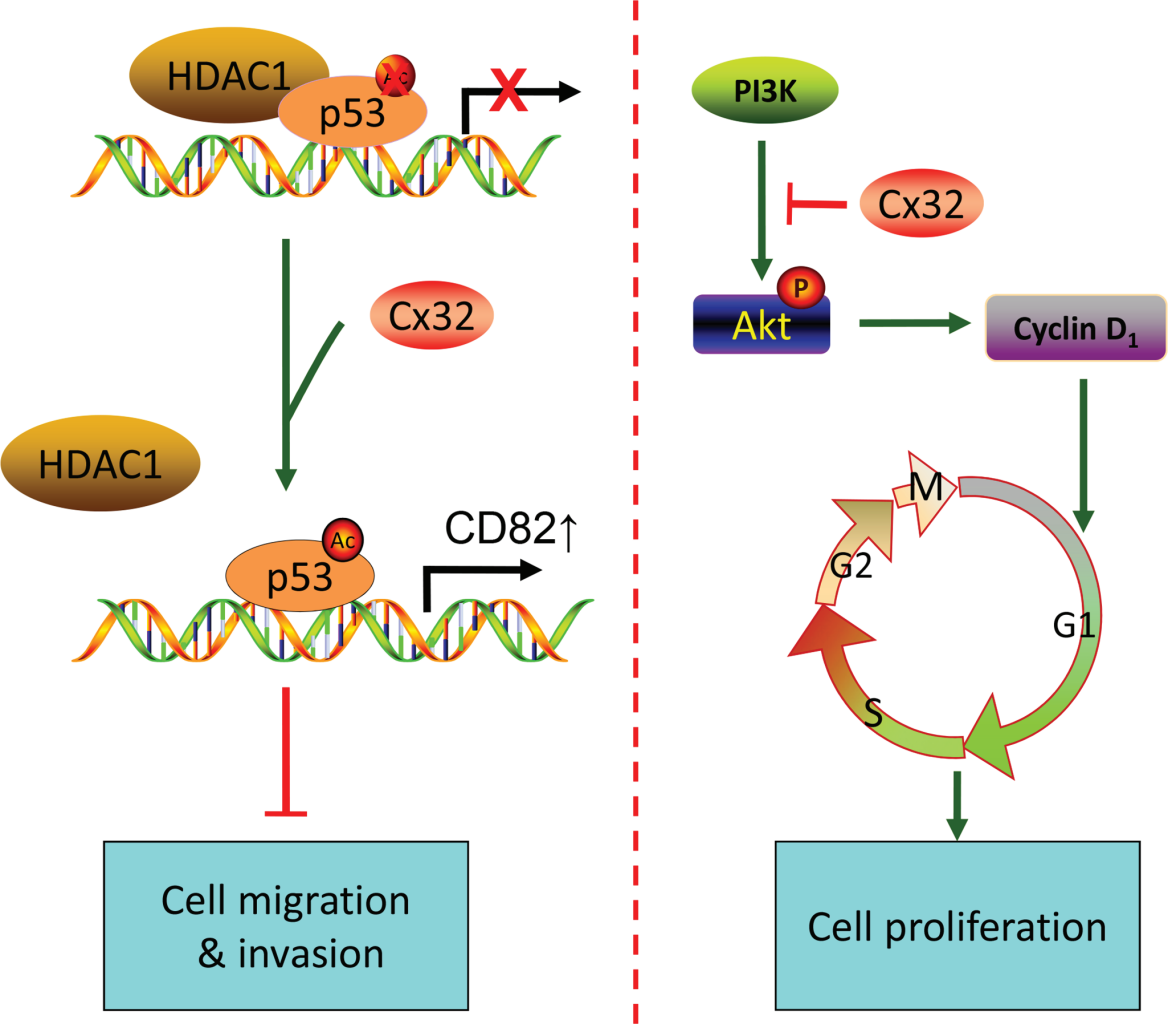
A proposed model for the mechanism by which Cx32 suppresses metastasis and proliferation of HCC cells through the p53 and Akt pathways, respectively

It is known that Cx genes act as tumor suppressors by the maintenance of cellular homeostasis via gap junctional intercellular communication (GJIC). A growing amount of evidence has accumulated, suggesting that at least in some cases, Cxs exert their tumor suppressive effects through a gap junction channel-independent pathway [28]. In many cases, re-expression of Cxs in both non-metastatic and metastatic tumor cell lines results in decreased cell proliferation and tumorigenesis and promotes favorable mesenchymal to epithelial-like transitions [29, 30]. In this study, we showed that Cx32 suppressed HCC proliferation and invasion, based on observations from human specimens, as well as from *in vitro* assays. We presented evidence supporting the notion that the downregulation of Cx32 in human HCC tissues is associated with enhanced vascular invasion and larger tumor size. *In vitro* studies showed that depletion or overexpression of Cx32 in HCC exhibited a significant inhibitory effect of Cx32 on cell invasion, migration, and proliferation. Furthermore, we identified the p53 and Akt signaling pathways as two novel targets that are partly responsible for the anti-metastatic and anti-proliferative functions of Cx32. The findings of this study suggested a crucial inhibitory function of Cx32 in tumor migration, invasion, and proliferation (Fig. 6).

Our study further confirmed the role of Cx32 as an anti-invasive gene in HCC cells with a wild-type p53 background. These findings are consistent with those of numerous previous reports documenting the anti-invasive role of Cxs in glioma stem cells [31] and renal cell carcinoma [32]. However, Cxs are not consistently identified as tumor suppressors, and these proteins can facilitate tumor progression during late-stage disease. Several reports suggest that Cxs might facilitate invasion and metastasis in human Huh7 HCC cells [11] and melanoma cells [33]. This leads one to consider the possibility that Cxs are differentially regulated during the development of specific tumor types. The role of Cxs in tumor cell invasion and metastasis seems to be complex and seems to depend on the different tumor types, Cx isoforms, and tumor progression stages. Even in the same type of cancer, Cx32 displays different functions in different cell lines. In a previous study of Cx32 in Huh7 cells, Cx32 facilitated invasion and metastasis [11], while in our study, an opposite effect of Cx32 on invasion and metastasis was demonstrated in HepG2 and SMMC-7721 cells.

In our study, p53 was a key regulatory protein involved in Cx32 function. In both the HepG2 and SMMC-7721 cell lines that harbor wild-type p53, Cx32 exerted an anti-invasive effect that was mediated by p53. However, Huh7 cells harbor a Y220C mutant p53 that is inactive, instead of a wild-type p53 [34]; thus, Cx32 may regulate cell invasion and metastasis through different mechanisms. Our study also indicated that in p53-null Hep3B cells, Cx32 alone could weakly inhibit cell migration; these data demonstrated that the p53 pathway was partly responsible for the anti-metastatic function of Cx32. However, the detailed mechanism remains to be elucidated.

The involvement of Cxs in the regulation of tumor cell proliferation has been suggested by a number of recent studies. One study, in which Cx32 expression was depleted in rat hepatoma cells, showed that the magnitude of cell proliferation is inversely proportional to the level of Cx32 expression [15]. Another study on gastric cancer showed that Cx32 inhibits gastric cancer cell proliferation through cell cycle arrest and altered expression of p21 and p27 [16]. The results of our *in vitro* Cx32 overexpression and knockdown studies are in agreement with these results. The mechanisms by which Cxs regulate proliferation appear to be more complicated. Cx43 has been shown to modulate the expression of several genes involved in the cell cycle, including cyclin A, cyclin D1, and cyclin D2, the cyclin-dependent kinases [35], and p21 and p27 [36]. Cx32 may also regulate cell cycle progression via a different pathway.

Our study showed a positive effect of Cx32 on the p53 signaling pathway and a negative effect of Cx32 on the Akt signaling pathway. Whether cross-talk exists between these two signaling pathways in the regulation of Cx32 warrants further exploration. It has been shown that p53 inhibits the Akt/mTOR pathway [37, 38]; we hypothesize that, when activated by Cx32, p53 not only transcriptionally activates KAI1/CD82, but also inhibits the Akt/mTOR pathway, thereby suppressing HCC cell proliferation and metastasis. It has been shown that microRNA-137 suppresses tumor growth and metastasis in human hepatocellular carcinoma by targeting AKT2 [39]. On the other hand, the negative effect of Cx32 on the Akt signaling pathway may also affect metastasis of HCC more than proliferation.

In our study, Cx32 activated p53 and inhibited proliferation of HCC cells. It is surprising that the expression of p21, a p53 target gene and a potent cyclin-dependent kinase inhibitor, was also downregulated by Cx32. This phenomenon is in contrast to the findings reported in previous relevant studies [16, 36]. We offer the following explanation for this discrepancy. First, p21 is not involved in the inhibitory function of Cx32 on HCC cell proliferation. Second, the inhibitory effect of Cx32 at the p21 protein level does not occur at the transcriptional level, but occurs instead at a post-transcriptional level. Cx32 inhibits the activity of Akt, and it has been reported that Akt phosphorylation of p21^Cip/WAF1^ enhances the stability of p21^Cip/WAF1^ and promotes cell survival [40]. Additionally, p21 exerts both positive and negative regulatory effects on cell cycle progression and is a regulator of cell survival. Finally, it is possible that Cx32 decreases p21 protein stability via Akt. Thus, the regulation of p21 expression by Cxs might vary according to the tumor type and Cx isoform. Additional studies are needed to elucidate Cx-mediated p21 expression mechanisms in HCC.

In conclusion, our study highlights the crucial role played by Cx32 in suppressing the progression of human HCC by inhibiting cell proliferation and invasiveness. Our data suggest that modulation of Cx32 could represent a future therapeutic strategy for the treatment of HCC.

## MATERIALS AND METHODS

### Cell lines and animals

The HCC cell lines HepG2, QGY-7701, SMMC-7721, and Hep3B and the human embryonic kidney cell line HEK 293T were purchased from the cell bank of the Shanghai Institute of Cell Biology (Shanghai, China). The MHCC97-H cell line was obtained from the Shanghai Cancer Institute. Cells were cultured in Dulbecco's modified Eagle's medium (DMEM; Gibco, Invitrogen), supplemented with 10% fetal bovine serum (FBS; HyClone), 100 U/ml penicillin, and 100 U/ml streptomycin. BALB/c nu/nu mice were purchased from the National Rodent Laboratory Animal Resources, Shanghai branch. Mice were kept in pathogen-free conditions and cared for according to the Laboratory Animal Care guidelines. Adult male animals, aged 8 to 10 weeks, were used. All the experimental protocols were reviewed and approved by our institutional review board. All animal experimental protocols were approved by the Institutional Animal Care and Use Committee of Xiamen University.

### Human tissue specimens

Tumor samples were obtained with informed consent from HCC patients at Zhongshan Hospital Xiamen University, between 2007 and 2011. The study was approved by the Xiamen University Medical Ethics Committee. Overall survival was calculated from the date of surgery to the date of death or final follow-up.

### Vector construction

The pcPUR+U6icassette plasmid, which contained an RNA interference sequence that targeted Cx32, was constructed as previously described [41]. Briefly, forward and reverse short-hairpin RNA (Table 2) that targeted Cx32 were annealed together and inserted into the *Bsp*MI site of the pcPUR+U6icassette vector, thereby generating the pcPUR+U6-siCx32 plasmid.

**Table 2:** siRNAs targeting the Cx32 gene

Site 1	Sense-oligo: 5′CACCCCGGCATTCTACTGCCATTACGTGTGCTGTCCGTAATGGCAGTAGAATGCCGG TTTTT 3′ Antisense-oligo: 5′GCATAAAAACCGGCATTCTACTGCCATTACGGACAGCACACGTAATGGCAGTAGAATGCCGG 3′
Site 2	Sense-oligo: 5′CACCGCTGCAACAGCGTTTGCTAACGTGTGCTGTCCGTTAGCAAACGCTGTTGCAGCTTTTT 3′ Antisense-oligo: 5′GCATAAAAAGCTGCAACAGCGTTTGCTAACGGACAGCACACGTTAGCAAACGCTGTTGCAGC 3′
Site 3	Sense-oligo: 5′CACCGGCTCACCAGCAACACATAACGTGTGCTGTCCGTTATGTGTTGCTGGTGAGCC TTTTT 3′ Antisense-oligo: 5′GCATAAAAAGGCTCACCAGCAACACATAACGGACAGCACACGTTATGTGTTGCTGGTGAGCC 3′

### RNA interference and stable cell lines

Small interfering RNA duplexes were purchased from RiboBio (Guangzhou, China). siRNAs were transfected using the HiPerFect siRNA transfection reagent (Qiagen), according to the manufacturer's instructions. To generate stably transfected cells, HepG2 or MHCC97H cells were transfected with pcPUR+U6-siCx32 or with control vectors by using the TurboFect transfection reagent (Fermentas) and stably transfected cells were established by treatment with 2 μg/ml puromycin for 3 weeks. The stable clones transfected with pcPUR+U6-siCx32 or pcPUR+U6 (control vector) are referred to as “shCx32” or “shCtrl,” respectively.

### RNA extraction and real-time PCR

Total RNA was extracted using the TRIzol reagent (Invitrogen, Carlsbad, CA), according to the manufacturer's instructions. Total RNA was reverse transcribed using the Prime Script TM RT reagent kit (Takara Biotechnology Dalian, China), according to the manufacturer's instructions. Quantitative real-time PCR was performed using the Maxima SYBR Green qPCR Master Mix (Fermentas). All values were normalized to the expression of the house-keeping gene glyceraldehyde-3-phosphate dehydrogenase (GAPDH), and the relative expression was calculated according to the 2^−ΔC(t)^ method: [ΔC_T_ = C_T_(Cx32)−C_T_(GAPDH)]. The following primers were used: Cx32-F: TGTCATCAGCGTGGTGTTC, Cx32-R: TTGCGGGAAGGTGGATTG; GAPDH-F: TGGCAAAGTGGAGATTGTTGCC, GAPDH-R: AAGATGGTGATGGGCTTCCCG.

### Western blot analysis

Equal amounts of protein lysates were separated by SDS-PAGE and were transferred onto PVDF membranes. The filters were probed with the following specific primary antibodies: Cx32 (Thermo), green fluorescent protein (GFP; Santa Cruz Biotechnology), histone deacetylase 1 (HDAC1; Proteintech), matrix metallopeptidase 2 (MMP2; Epitomics), vascular endothelial growth factor (VEGF; Thermo), proliferating cell nuclear antigen (PCNA; Abcam), beta-actin (Sigma), acetylated-p53 (Upstate Biotechnology), p53, Akt, phosphorylated-Akt, cyclin D1, and p21^Cip1/Waf1^ (Cell Signaling Technology). The blots were then incubated with horseradish peroxidase-conjugated secondary antibodies (Pierce) and visualized by chemiluminescence.

### Immunohistochemistry

Paraffin-embedded tissue samples were serially sectioned and immunohistochemically examined using an immunohistochemical staining kit (Maixin Bio, China), according to the manufacturer's instructions, with antibodies against Cx32 (Thermo).

### Luciferase reporter assay

The cells were transfected with a p53-luciferase reporter plasmid, β-galactosidase (β-gal), and additional expression vectors, as required. After transfection, cell lysates were prepared using the luciferase cell lysis buffer, and the luciferase and β-gal activities were measured. Luciferase activity was normalized to the transfection efficiency by using the corresponding β-gal activity. The ratios of the luciferase/β-gal activity were used as indicators of the transcriptional activity of the p53 promoter. The bars represent the mean ± SEM from three independent experiments.

### Tumor cell migration and invasion assays

Cell migration was assayed using the transwell method, with 8-μm pore filters (Corning, NY). The lower chamber was filled with Dulbecco's modified Eagle's medium (DMEM), supplemented with 10% fetal bovine serum (FBS), and 2 × 10^4^ cells in 0.5 ml of DMEM were loaded into the upper chamber. After a 22-hour incubation period, the cells that migrated to the bottom of the membrane were fixed with 4% formaldehyde. The cells on the top of the membrane were removed by wiping the surface with a cotton swab. The cells were stained with 0.5% crystal violet and observed under a microscope. The number of migrated cells was counted at a magnification of 200× from five adjacent microscope fields. For the Matrigel invasion assay, the procedures used were the same as those described above, except that the transwell membrane was coated with Matrigel (BD, CA, USA) to form a matrix barrier.

### EdU assay

The EdU incorporation assay was performed using the EdU assay kit (RiboBio, Guangzhou, China), according to the manufacturer's instructions. Briefly, SMMC-7721 cells were cultured in triplicate in 24-well plates and transfected with the Cx32 vector for 36 h. The cells were then incubated with 50 nM EdU for an additional 2 h at 37°C. The cells were fixed with 4% formaldehyde for 15 min at room temperature and treated with 0.5% Triton X-100 for 20 min at room temperature to permeabilize them. After three washes with phosphate-buffered saline (PBS), the cells were incubated with the Apollo reaction cocktail (100 μl/well) for 30 min. The DNA was stained with 10 μg/ml of Hoechst 33342 dye (100 μl/well) for 20 min and visualized with a fluorescence microscope.

### Wound healing assay

The cells were transfected and grown in 6-well plates until they reached 100% confluence. Migration ability was assessed by measuring the movement of cells into a scraped, acellular area that was created by a sterile pipet tip. Wound closure was observed after 24 h and was photographed under a microscope. The fraction of cell coverage across the line represents the migration rate.

### *In vivo* assays for tumor growth and metastasis

MHCC97H-shCtrl and MHCC97H-shCx32 cells (5 × 10^6^) in 0.2 ml of serum-free culture medium were inoculated subcutaneously into the right side of the backs of the nude mice. After a tumor formed, the tumor size was estimated according to the formula: volume (mm^3^) = a^2^ × b/2, where “a” is the major diameter of the tumor and “b” is the minor diameter perpendicular to the major one [42]. Eight weeks later, the animals were sacrificed. The tumors were removed and cryopreserved at −70°C for western blot analysis. Lungs were removed and embedded in paraffin for hematoxylin and eosin (H&E) staining.

### Co-IP assay

Cells were lysed in lysis buffer supplemented with protease inhibitors. Immunoprecipitation and western blot analyses were performed as previously described [43]. Briefly, the cell lysate was incubated with the appropriate antibody and protein A-agarose beads for 3 h. The immunoprecipitate was collected, washed three times with lysis buffer, and examined using western blot analysis with different antibodies, as required, after separation by SDS-PAGE. The immunoreactive products were visualized via enhanced chemiluminescence.

### Statistical analyses

The data were analyzed using GraphPad Prism, version 5 (GraphPad Software, Inc., San Diego, CA). The results are expressed as the mean ± SEM. Statistical analyses of normally distributed variables were performed using the Student's *t*-test, and analyses of data with skewed distributions were performed using the Mann-Whitney *U*-test. The relationship between the overall survival time and the Cx32 level was analyzed with Kaplan-Meier survival curves and the log-rank test. *p* < 0.05 was considered statistically significant.
